# Development and Validation of a Method for Profiling Post-Translational Modification Activities Using Protein Microarrays

**DOI:** 10.1371/journal.pone.0011332

**Published:** 2010-06-28

**Authors:** Sonia V. del Rincón, Jeff Rogers, Martin Widschwendter, Dahui Sun, Hans B. Sieburg, Charles Spruck

**Affiliations:** 1 Signal Transduction Program, Sanford-Burnham Medical Research Institute, La Jolla, California, United States of America; 2 Life Technologies Corporation, Carlsbad, California, United States of America; 3 Department of Gynaecological Oncology, University College London, London, United Kingdom; City of Hope National Medical Center, United States of America

## Abstract

**Background:**

Post-translational modifications (PTMs) impact on the stability, cellular location, and function of a protein thereby achieving a greater functional diversity of the proteome. To fully appreciate how PTMs modulate signaling networks, proteome-wide studies are necessary. However, the evaluation of PTMs on a proteome-wide scale has proven to be technically difficult. To facilitate these analyses we have developed a protein microarray-based assay that is capable of profiling PTM activities in complex biological mixtures such as whole-cell extracts and pathological specimens.

**Methodology/Principal Findings:**

In our assay, protein microarrays serve as a substrate platform for *in vitro* enzymatic reactions in which a recombinant ligase, or extracts prepared from whole cells or a pathological specimen is overlaid. The reactions include labeled modifiers (e.g., ubiquitin, SUMO1, or NEDD8), ATP regenerating system, and other required components (depending on the assay) that support the conjugation of the modifier. In this report, we apply this methodology to profile three molecularly complex PTMs (ubiquitylation, SUMOylation, and NEDDylation) using purified ligase enzymes and extracts prepared from cultured cell lines and pathological specimens. We further validate this approach by confirming the *in vivo* modification of several novel PTM substrates identified by our assay.

**Conclusions/Significance:**

This methodology offers several advantages over currently used PTM detection methods including ease of use, rapidity, scale, and sample source diversity. Furthermore, by allowing for the intrinsic enzymatic activities of cell populations or pathological states to be directly compared, this methodology could have widespread applications for the study of PTMs in human diseases and has the potential to be directly applied to most, if not all, basic PTM research.

## Introduction

Post-translational modifications (PTMs) are essential for the proper function of many proteins and dysregulation of these processes is known to play a causative role in several human diseases (reviewed in [1]). Modifications ranging from the simple conjugation of a phosphate group to the complex addition of ubiquitin can drastically alter the function of a protein. For example, the conjugation of ubiquitin to a substrate can modulate its activity, target it for degradation, alter its cellular location, or determine its interaction with other proteins [2]. Despite the importance of these modifications in maintaining cellular homeostasis and contribution to human diseases, identifying which proteins are modified by PTMs in mammalian cells on a proteome-wide scale has proven technically difficult. Moreover, methodologies for global proteomic analyses remain in their infancy due in large part to challenges encountered with developing proteomic platforms aimed at providing insight into basic biological processes [3], [4].

To overcome these technical limitations, we explored the possibility of using protein microarrays as a platform for profiling PTM activities. To date, the analysis of PTMs using protein microarrays has been somewhat limited to the phospho-proteome, profiling substrates of purified yeast enzymes, and characterizing substrates of the anaphase-promoting complex (APC) ubiquitin ligase [5], [6], [7], [8]. Phosphorylation is a ‘simple’ PTM compared to the complex enzymatic cascades required for many other modifications such as the conjugation of ubiquitin and ubiquitin-like (Ubl) modifiers (*e.g*. SUMO1 and NEDD8). These modifications are mediated by multi-step enzymatic reactions involving an activating (E1), conjugating (E2), and ligase (E3) enzyme that function consecutively to selectively transfer the PTM to substrates [1]. In this report, we describe a protein microarray-based methodology that is capable of profiling the ubiquitin and Ubl conjugation activities of recombinant ligases, cellular fractions, whole-cell extracts, and archival pathological specimens. We further apply this methodology to 1) identify novel substrates of the SCF^Skp2^ ubiquitin ligase, 2) profile for substrates of ubiquitylation, NEDDylation, and SUMOylation activities in whole-cell extracts, and 3) identify distinct changes in ubiquitin activity that associate with human tumor progression.

## Results

### Optimization of a protein microarray-based method to profile PTM activities

**Figure 1:**
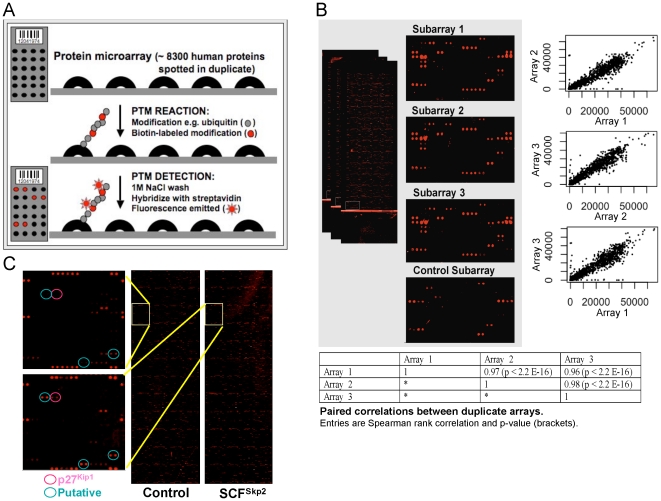
PTM profiling on protein microarrays. (A) Schematic of protein microarray-based profiling of PTM activities. Protein microarrays which display >8,000 recombinant human proteins spotted onto nitrocellulose-coated glass slides (in duplicate) provide a platform for assaying PTM activity. Reactions are performed ‘on-chip’ using purified enzymes or extracts prepared from cells or a pathological specimen, ATP regenerating system, modifier, and labeled-modifier (*e.g.* biotin-ubiquitin). PTM conjugation is then detected by incubating the slide with fluorescent-labeled ‘binders’ (*e.g.* streptavidin or antibodies) and activity quantified using a fluorescence slide reader. (B) Platform reproducibility. Shown are the results of three protein microarrays ubiquitylated in separate experiments and enlarged region of the protein microarray is shown on the left. Also shown are three pair-wise scatter plots that plot the signal intensity of each protein for the three biological replicates on the right. Statistical analysis of the data is shown below. (C) Profiling substrates of the SCF^Skp2^ ubiquitin ligase. Purified recombinant SCF^Skp2^ complexes were applied to protein microarrays in the presence of required co-factors (cyclin A-Cdk2 and Cks1). Insets show ubiquitylation of SCF^Skp2^ substrate p27^Kip1^ (red circle) and novel substrates (blue circles). SCF core (ligase complex minus the Skp2 substrate recognition component) was used as control.

A schematic of our methodology is shown in Figure 1A. Biochemical reactions are performed ‘on-chip’ by overlaying the protein microarrays with a purified conjugating enzyme or extract prepared from a biological specimen (*e.g.* cell line or pathological specimen) and all required co-factors. The protein microarrays are spotted with >8,000 different human recombinant proteins in duplicate which serve as substrates for PTM conjugation. The substrates are subsequently ‘tagged’ by conjugation of a labeled-modifier (*e.g.* biotin) present in the reaction mixture. Following a stringent wash to remove non-covalent substrate-modifier interactions, the PTM-conjugated substrates are then detected using ‘binders’ (*e.g.* antibodies or streptavidin) labeled with fluorescent dyes and the protein microarrays analyzed using a fluorescence slide scanner.

We first tried various configurations of ubiquitylation reactions using cellular fractions (S-100) and rabbit reticulocyte lysate to determine the optimal assay conditions. We evaluated different slide surface chemistries, reaction buffers, assay conditions, and detection methods. PATH slides (glass slides coated with nitrocellulose) proved to be superior to epoxy or hydragel-coated slides in reducing background (data not shown). The addition of 0.1% Tween-20 to both the reaction and wash buffers also significantly limited background and did not adversely affect PTM conjugation activity. Furthermore, the addition of inhibitors of de-conjugating enzymes (*e.g*. ubiquitin-aldehyde) to the reactions was found to increase PTM conjugation activity (data not shown). Moreover, washing the protein microarrays with 1 M NaCl +0.1% Tween-20 in PBS appeared to be sufficient for removing the non-covalent binding of modifiers to substrates since washes with 8 M urea, which is known to reduce non-covalent ubiquitin binding, was found to give an identical conjugation profile (data not shown). Although the use of protein microarrays to detect substrates of ubiquitylation has been previously reported [7], [8], we evaluated the reproducibility of our optimized ‘on-chip’ ubiquitylation reactions by statistically analyzing the results of three independent ubiquitylation reactions using whole-cell HeLa cell extract. Figure 1B shows an enlarged view of the same sub-array region from each of the three protein microarray replicates, wherein those spots producing fluorescent signals over background were found to be present on all three sub-arrays. Statistical analysis of the pair-wise scatter plots, plotting the signal intensity of each protein for each biological replicate, revealed a high degree of reproducibility between experiments (p<2.2 E-16; Fig. 1B).

### Identifying substrates of purified SCF^Skp2^ ubiquitin ligase

As proof of principle, we first sought to determine whether our assay system could be used to faithfully identify substrates of a purified E3 ubiquitin ligase. For these experiments, we utilized the ubiquitin ligase SCF^Skp2^ which has a well-defined role in human tumorigenesis [9]. SCF^Skp2^ is known to ubiquitylate several different substrates including the cyclin-dependent kinase (Cdk) inhibitor p27^Kip1^ [10], [11]. This reaction is molecularly complex and requires 1) substrate phosphorylation, 2) association of the substrate with cyclin A-Cdk2 complexes, and 3) the co-factor protein Cks1. We performed ‘on-chip’ ubiquitylation reactions that included recombinant human SCF^Skp2^ isolated from Sf9 insect cells, purified E1 and E2 enzymes, ATP regeneration system, ubiquitin, and biotin-labeled ubiquitin. The results of these experiments showed that p27^Kip1^ could be efficiently ubiquitylated on the protein microarray by SCF^Skp2^, and ubiquitin conjugation activity was only present when all the required components were added to the mixture, recapitulating the reaction conditions *in vivo* (Fig. 1C). In addition to p27^Kip1^, we identified several novel substrates of SCF^Skp2^ (Fig. 1C; see also **Substrate validation section**).

### Ubiquitylation reactions using cellular extracts

We next sought to determine whether this methodology could be used to accurately profile the PTM activity of complex biological mixtures, such as cellular extracts or pathological specimens. Using a 2-fold change as a cutoff over negative controls that lacked cellular extract, ubiquitylation reactions performed with rabbit reticulocyte lysate and S-100 fraction of HeLa cells revealed robust conjugation activities with 239 and 119 substrates identified, respectively (Table 1). Sixty-six substrates were found to be common to both the rabbit reticulocyte lysate and HeLa S-100 fraction (Table 2). Of these substrates, several were previously shown to either bind ubiquitin (*e.g.* LIVIN [12], RNF4 [13], ZNF364 [14]), contain ubiquitin binding domains (*e.g.* CUED1C [15], RAD23A [16]), or were known substrates of ubiquitylation activity (*e.g.* FLT1 [17], JAK2 [18], INSR [19]), lending strong support that this methodology faithfully detects true substrates of ubiquitin conjugation activity. We next profiled whole-cell extracts prepared from various cultured cell lines of both human and mouse origin and found these extracts efficiently ubiquitylated many (>120) different substrates on the protein microarrays (Table 1; data not shown). Approximately half of these substrates were found to be consistently ubiquitylated by all the cellular extracts analyzed. Collectively, these results demonstrate that this methodology could be used to profile biologically relevant PTM activity in complex biological specimens of various species origins.

**Table 1:** Ubiquitylated substrates profiled using cell extracts and tumor samples.

BC066929^12^	CCDC55^1^	FGFR3^2^	LOC370014^1235^	OR1Q1^1^	RPL41^1^	TSPAN17^12345^
XM_375359^1^	CCDC97^1^	FGFR4^1^	LOC440295^1^	PAK1^4^	RPS6KA1^2^	TSPO^4^
ABI1^1^	CDC2^1^	FGR^123^	LOC51491^1^	PAK3^1^	RPS6KA4^145^	TTK^14^
ABL1^12^	CDIPT^1^	FLT1^14^	LOC51765^45^	PBK^1^	RPS6KA5^145^	TYRO3^14^
ACBD6^12^	CDK2/cyclinA^1^	FLT3^1234^	LOC55319^1^	PDAP1^1^	RPS6KB1^4^	UBADC1^12345^
ACVR1B^14^	CDK9/cyclinT1^1^	FLT4^14^	LOC645591^4^	PDCL^13^	SCGB1C1^4^	UBE2C^1^
AHCYL1^2^	CETN3^12^	FRK^14^	LOC83786^1^	PDGFRalpha^1234^	SCYE1^1^	UBE2E2^15^
ADRBK2^14^	CHEK1^1^	G3BP1^1^	LOC84714^4^	PELI1^1^	SDCCAG3^1^	UBE2H^25^
AFF4^1^	CHERP^4^	GABRA3^1^	LYN^123^	PFDN5^45^	SEPT1^1^	UBE2O^124^
AIM2^4^	CHKA^4^	GADD45G^12^	MAGEB1^1^	PIM1^125^	SEPT5^1^	UBE2S^125^
AKT1^2^	CHUK^1^	GBA^4^	MAP2^1^	PIM2^145^	SERPINA3^1^	UBE3A^1235^
ANKHD1^1^	CLK3^4^	GMNN^1^	MAP2K2^1^	PKN2^14^	SGK^45^	UBQLN2^2^
ANKRD13A^1235^	CNOT7^12^	GNGT1^4^	MAP2K3^145^	PLK1^14^	SGK3^4^	UBXD1^13^
ANKRD13D^12345^	COPE^2^	GRK4^145^	MAP2K6^1^	PLK3^14^	SGPL1^1^	UBXD8^1^
ANKS4B^1^	COPZ1^15^	GRK6^14^	MAP3K2^14^	POMZP3^1^	SH3BP5^1^	VRK3^1^
APOBEC4^1^	CSAG1^1^	GSDMDC1^12^	MAP3K9^1^	PRKCalpha^145^	SIP1^4^	WDFY1^4^
ARL6IP4^1^	CSF1R^123^	GSK3B^14^	MAP4K5^145^	PRKCgamma^1^	SLAIN2^3^	WDR1^1^
ASCC2^1^	CSNK1D^14^	GYG2^1^	MAPK11^12^	PRKCH^1^	SLC6A13^1^	WEE1^1^
ASMTL^4^	CSNK1E^1^	HCK^12^	MAPKAPK3^1^	PRKCI^1^	SMCR7^15^	WIBG^2^
ATF6^1^	CSNK1G1^14^	HGS^2^	MAPKAPK5^12^	PRKG2^14^	SPATS2^1^	YES1^123^
ATP6V1G1^1^	CSNK1G3^4^	HOMER2^13^	MARK2^1^	PRKX^14^	SPDEF^1^	YY1^2^
ATXN3^12345^	CSNK2A1^1^	HPCAL1^4^	MATK^1^	PRRG1^1235^	SRMS^4^	ZAP70^1^
AURKB^1^	CSNK2A2^14^	HPGD^1^	MERTK^14^	PSMD4^12345^	SRPK1^1^	ZMYM5^123^
BIN1^4^	CUEDC1^12345^	IFI44L^4^	MET^1^	PSRC1^1^	SRPK2^1^	ZNF313^1^
BIRC7^124^	CXorf48^2^	IGF1R^123^	MINK1^4^	PTK2^1^	SRPK3^1^	ZNF364^12345^
BLK^1^	DAPK1^4^	IKBKB^1^	MPG^1^	PTPN5^1^	STIP1^1^	ZNF434^4^
BMX^1^	DAPK2^1^	ING5^1^	MSRB3^4^	RAB20^1^	STK17A^14^	
BRAF^4^	DHX32^1^	INSR^14^	MST1R^14^	RABEP2^25^	STK22D^1^	
BTK^14^	DNAJB2^2^	INSRR^145^	MYL5^14^	RAD23A^12345^	STK25^1^	
C10orf97^123^	DNAJC8^14^	IRAK4^13^	MYLK2^14^	RAF1^4^	STK3^145^	
C11orf52^1^	DYRK3^14^	IRF3^1^	NAP1L2^1^	RASGRP3^12^	STK4^14^	
C11orf53^1^	EIF5^1^	IRS1^2^	NBPF1^4^	RASL11B^2^	STRAP^1^	
C1orf165^1^	EPHA1^12345^	ITK^1^	NDUFB6^4^	RBCK1^1^	SULF1^45^	
C1orf91^1^	EPHA2^4^	JAK2^145^	NECAP1^1^	RBM34^1^	TAOK2^145^	
C20orf11^1^	EPHA5^14^	JAK3^14^	NECAP2^1^	RET^14^	TAOK3^145^	
C2orf13^45^	EPHA8^14^	KDR^1234^	NEK1^14^	RHBDD1^2^	TARBP2^1^	
C9orf78^1^	EPHB3^4^	KIAA1900^1^	NEK2^1^	RIOK3^12^	TBK1^1^	
CACNB1^1^	EPHB4^13^	KIF2C^1^	NEK4^1^	RNF34^1345^	TCP11^45^	
CALCOCO1^2^	ERBB2^125^	KIF3B^1^	NEK6^1^	RNF111^12345^	TCP11L1^15^	
CAMK1^123^	ERBB4^4^	KIT^1^	NEK9^145^	RNF126^ 235^	TEC^1^	
CAMK1D^1^	FAM126B^2^	LCK^1^	NFKBIB^1^	RNF128 2	TEK^14^	
CAMK2N1^1^	FAM112B^1^	LMNA^1^	NGLY1^2^	RNF130 2	TMEM139^2^	
CAMK2N2^12^	FAM50A^1^	LOC10572^2^	NMT1^1^	RNF185^1235^	TNIK^1^	
CAMKIIalpha^1^	FES^1^	LOC112860^4^	NR4A1^1^	RNF4^1245^	TNIP2^125^	
CAMKIIdelta^1^	FER^4^	LOC115460^1^	NTRK1^1^	ROR1^1^	TOM1^125^	
CASQ2^1^	FGF21^2^	LOC120376^1^	NTRK2^1^	ROR2^45^	TOM1L2^12345^	
CAT^1^	FGFR1^12^	LOC121457^4^	NTRK3^1^	ROS1^1^	TRIM44^1^	
CCDC12^1^	FGFR2^1^	LOC284440^4^	NUAK1^1^	RPAIN^12^	TRIM52^12345^	

1 Rabbit reticulocyte lysates,

2 Mouse embryonic fibroblasts,

3 Human foreskin fibroblasts,

4 HeLa cell S-100 fractions,

5 Breast tumor specimens.

**Table 2:** Ubiquitin, NEDD8, and SUMO1 conjugated proteins identified on protein microarrays.

UBIQUITYLATION				NEDDYLATION		SUMOYLATION
UPS-associated		Ubiquitin Substrates		NEDD8 Substrates		SUMO1 Substrates
ACVR1B*	MST1R*	ADRBK2	MYLK2	ANKHD1	LSM3	*ADRBK1*
ATXN3 ^F^	PDGFRalpha* ^F^	ANKRD13D ^F^	NEK1	ANKRD13D	MAP3K10	*AKT2*
BTK*	PLK1*	CSNK1D	NEK9	ANKRD17	MAP3K11	CDK5
CAT*	PLK3*	CSNK1G1	**PIM2**	ANKRD39	MAP3K9	*CENPB*
CUEDC1 ^F^	PRKCalpha*	CSNK2A2	**PKN2**	ANKS4B	MATK	*COPE*
FLT1*	PRKCgamma*	DYRK3	PRKX	BTK	MCC	*FES*
FLT3*	PSMD4 ^F^	EPHA1	**ROS1**	CCDC69	MINK1	FGFR3
GSK3beta*	RAD23A ^F^	EPHA5	**RPS6KA4**	CENPB	MST1R	*FGR*
INSR*	RET*	**FLT4**	**RPS6KA5**	CETN3	NAP1L1	*FYN*
ITK	RNF4	FRK	STK3	CHEK1	NFKBIB	*HIPK3*
JAK2*	RNF111 ^F^	GRK4	STK4	CSNK2A1	OTUD6B	HK1
JAK3*	TTK*	GRK6	STK17A	CUEDC1	PAIP2	*ING3*
LIVIN	UBADC1 ^F^	INSRR	**TAOK2**	CXorf48	PAK1	JAK3
MAP3K2*	UBE2O	KIAA1900	**TAOK3**	DIXDC1	PAK3	*LCK*
MAP4K5*	ZNF364 ^F^	MAP2K3	TEK	EIF2B2	PBK	LENG4
		**MCAK**	**TRIM52**	EPHA1	PDCL	*MAPKAPK5*
		MERTK	**TSPAN17**	EPHB4	PEX19	*MERTK*
		MYL5	TYRO3	FAIM	PIM1	*PAK3*
				FGR	PRKCalpha	*PBK*
				GCC1	PRKCepsilon	*RBCK1*
				GOPC	PSCD1	*RIPK2*
				GSDMDC1	RAD23A	RNF4
				LCK	RGS20	*RPS6KA3*
				LGALS3	RPS6KB1	STK3
				LMNA	TOM1L2	*VPS29*
				LOC126382	TRIM44	ZMYM5
				LOC57596	UBOX5	

Substrates shown for ubiquitin are common to both rabbit reticulocyte lysate and HeLa S-100 fractions.

Underlined, E3-associated;

*known substrate of ubiquitylation;

**Bold**, high homology to proteins known to be ubiquitylated;

F Superscript, substrates also common to human fibroblasts;

*Italics*, SUMO1 substrates containing SUMO consensus sequences (yKxE/D). UPS, ubiquitin proteasome system.

### Profiling changes in ubiquitylation activity associated with human disease

**Figure 2:**
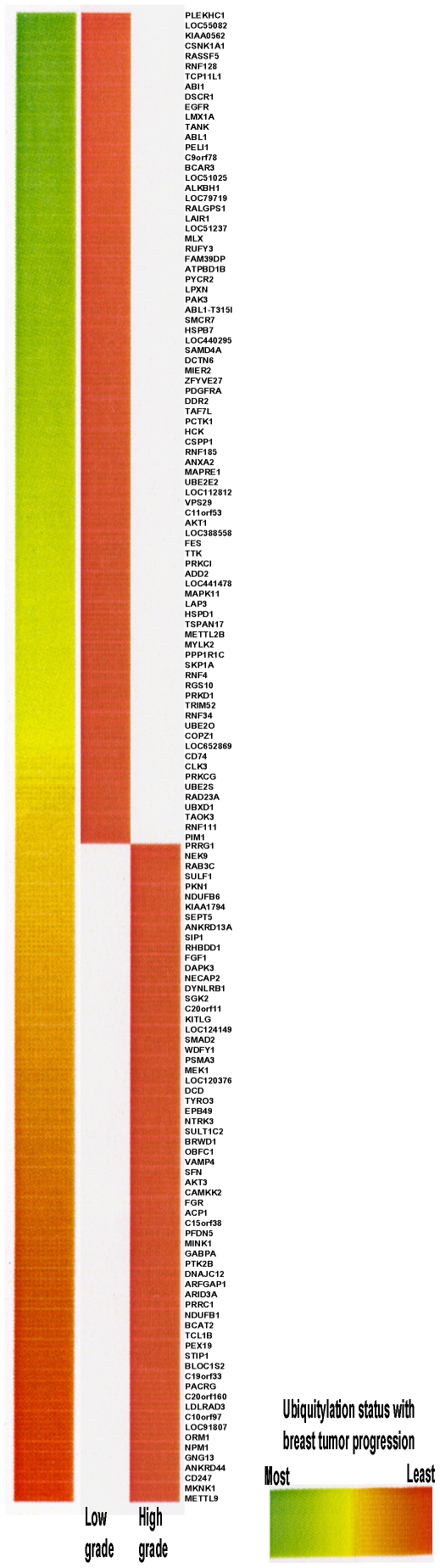
Comparison of ubiquitylation changes in low and high grade tumor samples by protein microarray analysis. Column 4 lists the protein names sorted according to a directional measure of fold-change in ubiquitylation status. Specifically, if the median measurement for low grade tumors exceeded the median value for high grade tumors we assigned a negative ratio of low/high. Otherwise, a positive ratio was assigned. The directional change is reflected in the heat map (Column 1), which shows the color distribution across a red (smallest-negative) to green (highest-positive) color spectrum. In the middle columns, the change of white to red signifies that high fluorescence values in reactions containing low grade tumor extract correspond to low fluorescence values in reactions containing high grade tumor lysate, that is, the protein is more ubiquitylated in low grade tumors compared to high grade tumors.

A clinically relevant application of this methodology is comparative profiling, wherein disease-associated changes in PTM activity are compared to the normal state. To this end, we applied this methodology to identify changes in ubiquitylation activity that occurs during the progression of human tumors to more advanced and life-threatening disease. Remarkably, we found that human breast tumor specimens that had been kept frozen at −80°C for >10 years contained robust ubiquitin conjugation activity (Table 1) comparable to that observed for cellular fractions or whole-cell extracts prepared from cultured cells. We next pooled extracts prepared from 5 low-grade and 5 high-grade breast tumors and performed ‘on-chip’ ubiquitylation reactions with these extracts. Using a 1.6-fold change as a cutoff over negative control reactions that lacked tumor lysate, we identified several differentially ubiquitylated substrates between the low-grade and high-grade specimens. These results are visually represented as a heat map in Figure 2 (fold changes are listed in Table S1). Interestingly, the majority of the differentially ubiquitylated substrates were found to have defined roles in several processes implicated in tumor progression. One of the proteins showing increased ubiquitylation in high-grade tumors was RAD23A [20], [21]. RAD23 is implicated in DNA repair and is known to interact with the E3 ligase E6AP, suggesting that its degradation by ubiquitylation may contribute to tumor progression through impairment of the DNA repair process. Moreover, TRIM52, a protein that possesses intrinsic E3 ubiquitin ligase activity, demonstrated increased ubiquitylation in high-grade tumors suggesting that it may also be targeted for degradation by ubiquitylation. In support of this, we found that TRIM52 is indeed a target of the ubiquitin proteasome pathway (see **Substrate validation section**).

### Protein microarray-based profiling of Ubl modifications SUMO1 and NEDD8

We next determined whether this methodology could be easily adapted to other complex PTMs, such as SUMO1 (small ubiquitin-like modifier 1) and NEDD8 (neural precursor cell expressed and developmentally down-regulated 8). SUMO1 and NEDD8 are conjugated to substrates in multi-step enzymatic reactions similar to but distinct from ubiquitylation [22]. Reaction conditions used in our assay were similar to those used for the conjugation to ubiquitin (described above) except for the substitution of the relevant reaction buffer, E1 enzyme, aldehyde derivative, and biotin-labeled modifier. The results of these experiments showed that HeLa cell extracts efficiently conjugated SUMO1 and NEDD8 to many substrates on the protein microarrays (Table 2). Of the putative SUMOylated substrates identified, HIPK3 [23] and RNF4 [13] were previously shown to bind SUMO1 and the majority of the remaining substrates contained consensus SUMO1 targeting sequences (yKxE/D) [24]. Although only a few substrates of NEDDylation have been reported in the literature [25], [26], [27], our screen did detect LGALS3, which was previously shown to be NEDDylated using an alternative proteomic approach [27]. Of note, we failed to detect NEDDylation of the well-known NEDD8 target cullin protein family with our assay (cullins 1, 3, 4a, and 4b are displayed on the protein microarrays but the level of conjugation activities did not meet our 2-fold cutoff criteria). This lack of activity could be due to a number of factors. Although it is readily accepted that cullins are NEDDylated on the Lys in the conserved sequence IVRIMKMR [28], the accessory factors required for promoting cullin NEDDylation may be molecularly complex and is still an area of active investigation. *In vitro* evidence shows that the RING finger protein Rbx1 is required for cullin NEDDylation [29], [30], [31], while *in vivo* NEDDylation is enhanced by DCN1 [32]. Moreover, the ability to detect cullin protein NEDDylated may be influenced by de-NEDDylase activities (*e.g*. COP9 Signalosome) [33]. Therefore, it is plausible that the activity of Rbx1 or DCN1 present in our reactions was limiting or de-NEDDylase activity was dominant in our assay. Alternatively, these proteins may not be appropriately folded or pre-modified in insect cells (used for recombinant protein expression) and cannot be appropriately recognized by the NEDDylation machinery using our reaction conditions.

### Substrate validation experiments

**Figure 3:**
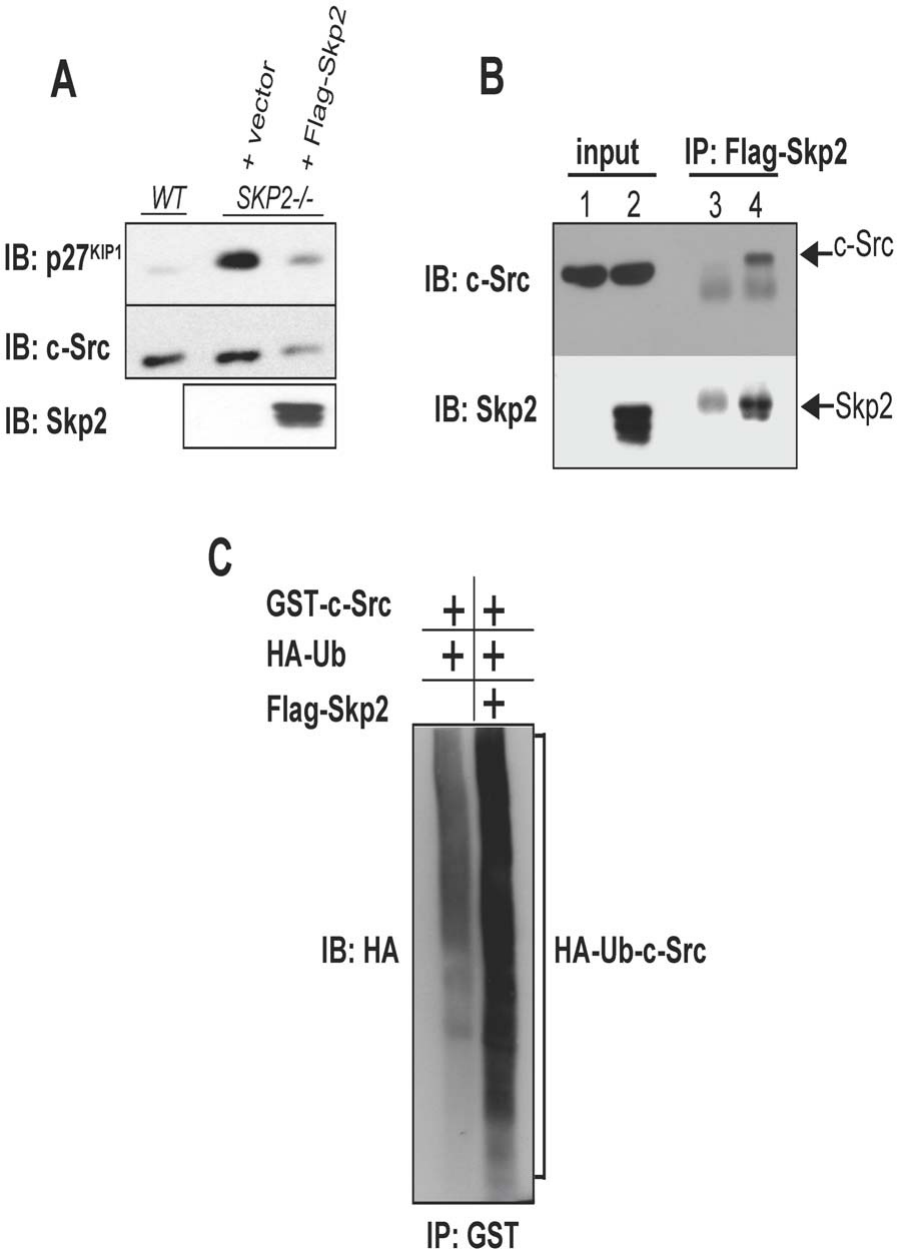
Validation of c-Src as a novel SCF^SKP2^ substrate. (A) *SKP2* ^−/−^ MEFs were transduced with control (pBABEpuro) or Flag-Skp2-expressing retroviruses and Western blot analysis was used to assess the expression level of known SCF^Skp2^ substrate p27^Kip1^ and putative substrate c-Src. (B) Endogenous c-Src associates with Skp2 *in vivo*. Anti-Flag antibodies were used to immunoprecipitate Flag-Skp2 from extracts prepared from *SKP2^−/−^* MEFs transduced with control (lanes 1 and 3) or Skp2-expressing retroviruses (lanes 2 and 4). Association of c-Src with Skp2 was determined by Western blot analysis. The same blot was then re-probed with anti-Skp2 antibodies to verify immunoprecipitation. (C) Skp2 promotes c-Src ubiquitylation *in vivo*. HEK293T cells were co-transfected with plasmids that express GST-c-Src, HA-Ubiquitin, with or without Flag-Skp2. Extracts from cells were denatured, c-Src immunoprecipitated using anti-GST antibodies, and ubiquitylation detected by Western blotting with anti-HA antibodies.

To determine the accuracy of our assay system in detecting true PTM conjugation activities, we first randomly selected c-Src, a SCF^Skp2^ substrate identified using our assay but not reported in the literature, and determined if it was indeed a substrate of SCF^Skp2^ *in vivo*. c-Src is a non-receptor tyrosine kinase that plays an important role in regulating cell proliferation and its augmented expression promotes tumor cell invasion and metastasis [34]. To validate c-Src as a novel SCF^Skp2^ substrate, we transduced *SKP2^−/−^* knockout MEFs with retroviruses that express Skp2 and found this induced the down-regulation of c-Src protein levels, consistent with its enforced degradation (Fig. 3A). Moreover, immunoprecipitation of Skp2 from these cell extracts revealed that endogenous c-Src associates with Skp2 *in vivo* (Fig. 3B). Furthermore, ectopic expression of Skp2 in HEK293T cells was found to stimulate c-Src ubiquitylation *in vivo* (Fig. 3C). Collectively, these results are consistent with SCF^Skp2^ regulating the degradation of c-Src through ubiquitin-dependent proteolysis.

**Figure 4:**
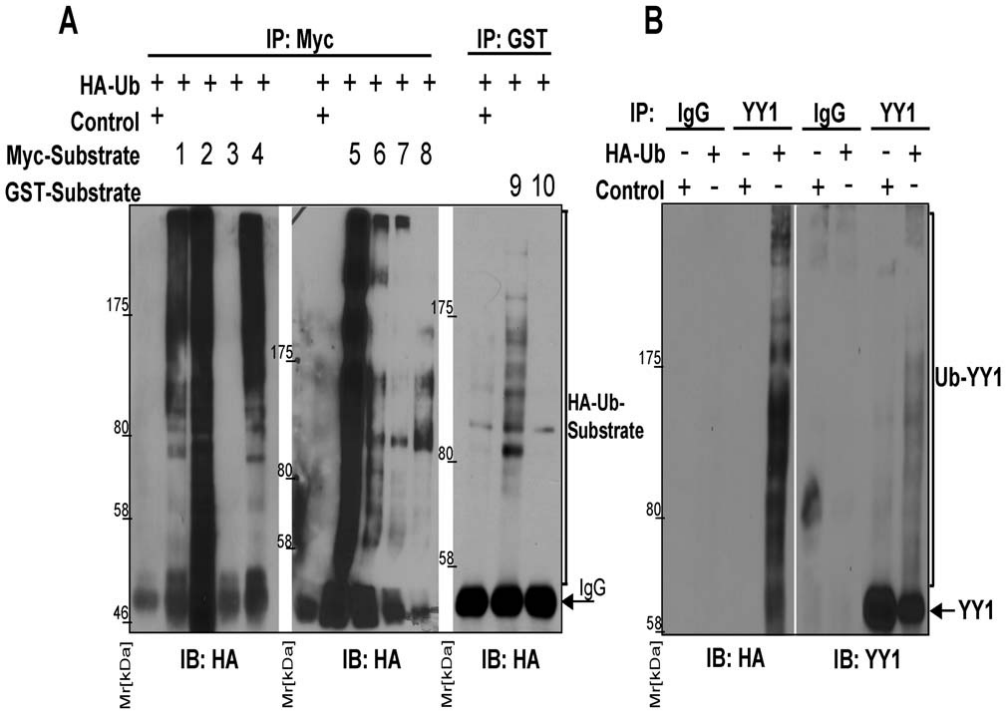
*In vivo* validation of substrates ubiquitylated on protein microarrays. (A) Ten putative substrates of ubiquitylation identified on the protein microarrays but not reported in the literature were selected for validation of the modification *in vivo*. Myc- or GST-substrates were co-expressed with HA-ubiquitin in HEK293T cells. HEK293T cell extracts were prepared using denaturing conditions, substrates immunoprecipitated with anti-Myc or anti-GST antibodies, and ubiquitylation detected by immunoblotting with anti-HA antibodies. Empty vector co-expressed with HA-tagged ubiquitin served as control. Substrates indicated in each lane are: 1- ADRBK2, 2- ACVR1B, 3- PIM2, 4- PRKCgamma, 5- KIF2C, 6- RPS6KA5, 7- ITK, 8- EPHA1, 9- TRIM52, and 10- EPHA5. Of 10 substrates 8 were found to be expressed and immunoprecipitated at detectable levels and of these all demonstrated evidence of ubiquitylation *in vivo*. To best visualize an ubiquitin smear, substrates 1, 2, 3, 4 were separated by 10% SDS-PAGE gels, while larger molecular weight substrates 5, 6, 7, 8 were separated by 6% SDS-PAGE gels. (B) Ubiquitylation of YY1. HEK293T cells were transfected with plasmids that express HA-ubiquitin, endogenous YY1 protein immunoprecipitated from the denatured extracts, and conjugation to ubiquitin determined by Western blot analysis with anti-HA antibodies (left). Immunoprecipitation efficiency was determined by probing blots with anti-YY1 antibodies (right). Immunoprecipitation with IgG antibodies of the same species served as control.

To further validate the accuracy of our methodology, we randomly selected 10 substrates which were shown to be ubiquitylated on the protein microarrays (by both rabbit reticulocyte lysate and HeLa S-100 fraction) but whose modification was not reported in the literature and attempted to verify whether they were substrates of ubiquitylation *in vivo*. HEK293T cells were co-transfected with plasmids that express HA-tagged ubiquitin and the Myc- or GST-tagged substrates activin A receptor-type IB (ACVR1B), beta-adrenergic receptor kinase 2 (ADRBK2), IL2-inducible T-cell kinase (ITK), protein kinase C-gamma (PRKCgamma), ephrin type-A receptor 1 (EPHA1), serine/threonine protein kinase PIM2, 90 kDa ribosomal protein S6 kinase 5 (RPS6KA5), kinesin family member 2C (KIF2C), ephrin type-A receptor 5 (EPHA5), or tripartite motif-containing protein 52 (TRIM52) (Fig. S1). To determine whether these substrates were covalently conjugated to ubiquitin *in vivo*, we subjected the HEK293T extracts to denaturing immunoprecipitation, which included lysis of cells in buffer containing 1% SDS and boiling the samples prior to immunoprecipitation [35]. Of the 8 substrates that were expressed and immunoprecipitated at detectable levels all were found to be ubiquitylated *in vivo* (Fig. 4A; data not shown). Bayesian statistical testing [36], [37] of these results verified that substrates that were ubiquitylated on the protein microarrays had a high-probability of being true substrates of ubiquitylation *in vivo* (the null hypothesis was tested H_0_∶ p = 0.5 against the probability P* = 0.63 derived from our validation data and rejected with evidence ev = 0.89). To confirm that the observed ubiquitylation *in vivo* was not due to substrate overexpression, we immunoprecipitated endogenous YY1 protein, a putative substrate of ubiquitylation identified by our assay and regulator of the MDM2 ubiquitin ligase that controls the ubiquitin-dependent proteolysis of p53 [38], from HEK293T cell extracts using denaturing conditions and analyzed its ubiquitylation status by Western blot analysis. These experiments clearly showed that endogenous YY1 was indeed ubiquitylated *in vivo* (Fig. 4B).

**Figure 5:**
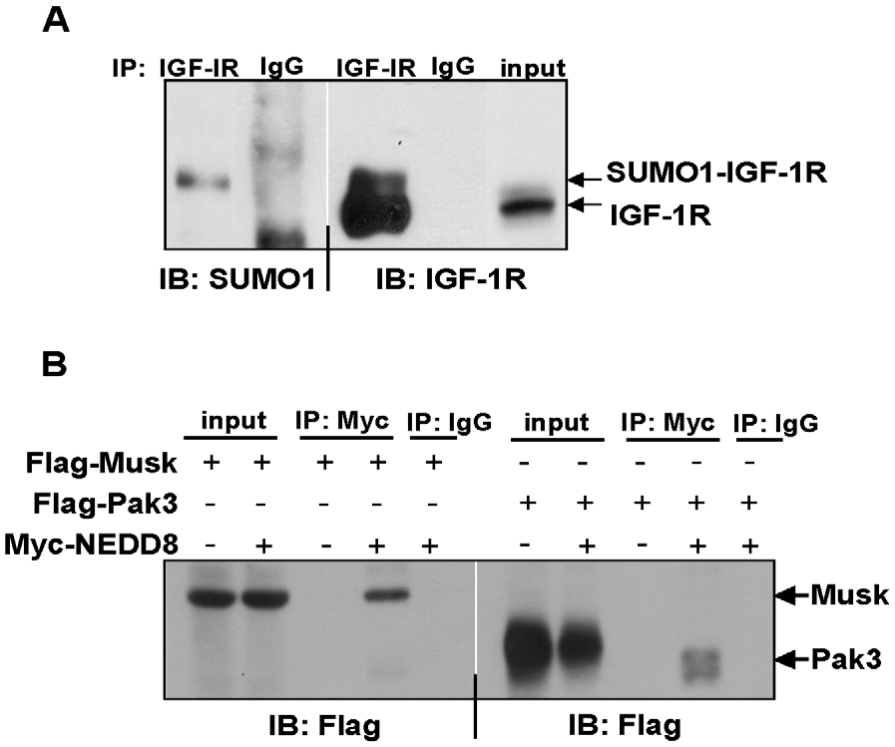
*In vivo* confirmation of SUMO1 and NEDD8 substrates identified on protein microarrays. (A) SUMOylation of IGF-1R. Endogenous IGF-1R was immunoprecipitated from denatured extracts prepared from HEK293T cells and conjugation to SUMO1 determined by Western blot analysis with anti-SUMO1 antibodies. Immunoprecipitation efficiency was determined by Western blotting with anti-IGF-1R antibodies (right). (B) NEDDylation of Musk and Pak3. HEK293T cells were transfected with plasmids that express Flag-Musk or Flag-Pak3 with or without Myc-NEDD8. Denatured extracts were then immunoprecipitated with anti-Myc or IgG antibodies (control) and conjugation to NEDD8 determined by Western blotting using anti-Flag antibodies.

We next tested the accuracy of our assay in profiling SUMO1 and NEDD8 conjugation activities using similar experimental strategies. Immunoprecipitation of endogenous insulin-like growth factor 1 receptor (IGF-1R), a receptor tyrosine kinase that mediates IGF1 signaling [38], from HEK293T cell extracts using denaturing conditions confirmed that it was covalently conjugated to SUMO1 *in vivo* (Fig. 5A). Furthermore, p21^Cip1^-activated kinase 3 (Pak3), which is associated with non-syndromic mental retardation in humans [39], and Musk, a receptor tyrosine kinase that plays a role in neuromuscular junction organization [40], were found to be covalently conjugated to NEDD8 *in vivo* (Fig. 5B).

## Discussion

The results of our analyses demonstrate that our protein microarray-based methodology can reliably and accurately profile PTM conjugation activities in simple (*e.g*. purified PTM ligases) and complex (*e.g*. whole-cell extracts) biological samples. The assay system is highly reproducible, sensitive (can be performed with as little as 2 µg of whole-cell extract), rapid (analysis can be completed in a single day), and can be easily adapted to profile a variety of different PTM conjugation activities. In this study, we used our assay to 1) identify novel substrates of the SCF^Skp2^ ubiquitin ligase, 2) profile ubiquitin, SUMO1, and NEDD8 conjugation activities of whole-cell extracts, and 3) define changes in ubiquitylation activity that associate with human breast tumor progression. As further validation of this methodology, during the preparation of this manuscript another group used a similar approach to identify novel substrates of the APC ubiquitin ligase [8].

Current techniques used to identify substrates of PTMs on a proteome-wide scale include two-hybrid and high-copy suppressor screens in yeast and mass spectrometry [27], [41], [42], [43]. However, these techniques have several limitations. For example, PTM analysis by proteomic mass spectrometry can be hindered by 1) low substrate abundance, a characteristic of many ubiquitylated proteins, and/or a sub-stoichiometric level of PTM, 2) the labile nature of many PTMs, making their preservation through biochemical purification, separation, fragmentation, and analysis problematic, especially if native conditions are required leaving substrates vulnerable to de-conjugating enzymes, 3) the adverse effects of certain PTMs on proteases, ionization, and detection efficiency, and 4) multi-site or multi-species modifications, which could make data interpretation problematic.

Our methodology overcomes many of these limitations and provides several advantages over these currently employed techniques. Since our assay relies on the intrinsic PTM conjugation activity of a specimen it is less sensitive to substrate concentrations and sub-stoichiometric modifications can be easily detected. The reactions can also be performed with crude extracts eliminating elaborate purification protocols that could promote de-conjugation of the PTMs. Furthermore, we have successfully multiplexed our assay system to simultaneously profile the conjugation activities of several different PTMs simultaneously on a single protein microarray using differentially labeled fluorescent antibodies for PTM detection (data not shown).

However, there are some potential limitations with our assay system. First, the protein microarrays used in this study display ∼8,000 human proteins, representing only ∼1/3 of the proteome. Secondly, since the protein microarrays are produced with recombinant human proteins expressed in Sf9 insect cells a proportion of these substrates could be misfolded, possibly precluding their modification or promoting their artificial modification. Thirdly, our methodology may underestimate the number of proteins post-translationally modified if the substrates are printed on the microarrays in a manner that masks a specific sequence that must be recognized by the PTM conjugating enzyme, such as the ubiquitin ligase APC/C^CDC20^ which uses a destruction box motif (termed D box) for recognition [44]. Another potential scenario for this underestimation could be that the arrayed proteins are pre-modified by the conjugation activity in insect cells prior to spotting on the protein microarrays. This may at least hold true for ubiquitylation, since there is evidence that exogenously expressed proteins in Sf9 insect cells can be ubiquitylated *in vivo* [45]. However, evidence suggests that even though they contain SUMOylation machinery, Sf9 cells cannot support SUMOylation of exogenously expressed human proteins [46]. Fourthly, being a purely *in vitro* assay, *in vivo* regulatory processes (*e.g.* temporal or spatial regulations) will likely be lost during extract preparation. Finally, information regarding the site of PTM attachment to a substrate cannot be ascertained. Therefore, our assay system might be most effective when it is used in conjunction with other screening techniques and any conjugation activities identified should be thoroughly validated *in vivo*.

Considering that dysfunction of PTMs play a critical role in a number of pathological states in humans, this methodology is an important step forward in the field of proteomics because it will allow for alterations of PTM activities associated with human diseases to be identified. For example, SUMOylation is known to play an important role in maintaining genomic integrity and preventing tumorigenesis. The SUMOylation machinery is recruited to sites of DNA damage, and both the tumor suppressor BRCA1 and the DNA repair factor 53BP1 are substrates of SUMOylation [47], [48], [49]. Our methodology could be used to further unravel the role of SUMOylation in the DNA damage repair process, such as through comparison profiling of SUMOylation activities from extracts prepared from UV-irradiated and control cells. A comparison of extracts from normal and cancer cells with defective DNA damage repair might also help to define how this process is dysregulated in cancers. Another example are the deubiquitylating enzymes (DUBs), which function to counteract the E3 ubiquitin ligases by removing ubiquitin from substrates and may play an important role in cancer. One such DUB is A20, which is an NFκB inhibitor and tumor suppressor [50]. However, the molecular substrates of A20 are largely unknown. Our methodology might be employed for these studies by incubating protein microarrays that were pre-ubiquitylated by cellular extracts with recombinant A20 protein and profiling for losses in substrate fluorescence.

In combination with genetic mutants, small molecule perturbants, or RNAi technology, our methodology could help to define both specific and global aspects of PTMs. Modified cell lines, disease model systems, and specialized tissues all lend themselves well to PTM profiling using this approach with the ultimate goal of furthering our understanding of disease states and identifying novel therapeutic targets for their treatment.

## Materials and Methods

### Protein microarrays

Several versions of the ProtoArray Human Protein Microarray (Invitrogen) were utilized in this study. Profiling experiments performed with purified ligases, whole-cell extracts, and tumor extracts utilized version 4 arrays. These protein microarrays display >8000 purified human proteins (in duplicate) on a nitrocellulose-coated glass slide. Each of the >8000 human proteins are derived from human open reading frames (ORF) that were expressed in Sf9 insect cells as an N-terminal GST fusion protein.

### Extract preparation

Cell lines (HeLa, mouse and human fibroblasts) and tumor (fresh-frozen human breast cancer tissue) specimens were suspended in lysis buffer (20 mM Hepes (pH 7.4), 2.5 mM MgCl_2_, 0.5 mM DTT, 5 mM NaF, 1 mM sodium orthovanadate, 1 mM PMSF, 2 µg/ml aprotinin, 1 µg/ml pepstatin, and 1 µg/ml leupeptin) on ice for 15 min and then sonicated briefly. The extracts were clarified by centrifugation for 15 min at 14,000× *g* and snap-frozen in liquid nitrogen until use. Rabbit reticulocyte lysate and HeLa S-100 fraction were purchased (Boston Biochem).

### Recombinant proteins

Human SCF^Skp2^ complexes were produced in Sf9 insect cells as described previously [11]. Recombinant Cks1 was produced in bacteria and purified as described [51]. Cyclin A-Cdk2 complexes were purchased (Life Technologies).

### Antibodies

Antibodies used in this study included: anti-ubiquitin (Biomol, PW8805); anti-SUMO1 (Zymed, 33-2400); anti-NEDD8 (Zymed, 34-1400); anti-p27^Kip1^ (BD pharmingen); anti-c-Src (Biosource); anti-Skp2 (Zymed), anti-YY1 (Santa Cruz Biotechnology); anti-IGF-1R (Zymed); anti-HA (Covance); anti-Flag (Sigma); anti-GST (Santa Cruz Biotechnology); and anti-Myc (9E10, Santa Cruz Biotechnology).

### PTM profiling

Extracts (2-100 µg in 40 µl of lysis buffer) were combined with either 4 µM of ubiquitin aldehyde (Boston Biochem) to prevent the action of deubiquitylating enzymes in the ubiquitylation reactions, SUMO1 aldehyde to inhibit SUMO-specific isopeptidases (SENPs) (Boston Biochem) in SUMOylation reactions, or NEDD8 aldehyde to inhibit deNEDDylating and NEDD8 processing enzymes in NEDDylation reactions (Boston Biochem), and then incubated at 25°C for 15 min. The reactions were then supplemented with modifier (1.25 µg/ml), biotin-labeled modifier (50 ng/ml), Tween-20 (0.1%), energy regenerating system (Boston Biochem), and 1× reaction buffer (ubiquitylation, SUMOylation, NEDDylation; Boston Biochem) in a final volume of 100 µl. Proteasome inhibitor MG132 (5 µM) was added to ubiquitylation reactions. For SCF^Skp2^ experiments, reaction conditions were as described [11]. The reaction mixtures were applied to the protein microarrays, covered with glass coverslips equipped with rubber gaskets to avoid leakage (Life Technologies), and then incubated at 37°C for 1 hr in a humidified chamber. The arrays were then washed in PBS-Tween (0.1%, PBST) containing 1 M NaCl for 10 min, 2×10 min in PBST, and then incubated with Streptavidin Alexa Fluor 647 (100 ng/ml; Life Technologies) for 1 hr at 25°C. The arrays were then washed 3×10 min in PBST and spun dry. Imaging was performed using a GenePix 4000B Slide Imager (Molecular Devices) and fluorescent spots analyzed using GenePix Pro software. Gal files (which contain array production information and spot location, identification, and quantification) were downloaded from www.invitrogen.com and used with GenePix Pro software to analyze the median intensity of each spot. All data evaluations were done using the statistical program R [52]. Specifically, we first filtered the data with a cutoff threshold of 5000 counts for the fluorescence values, and then applied the Benjamini-Hochberg procedure [53] with control of the false discovery rate (FDR) set at the 5% level. The resulting set of proteins was used to mine UniProt and PubMed using the BioConductor modules of R.

### Substrate validation experiments

*SKP2^−/−^* MEFs were transduced with control (pBabe) or 3× FLAG-Skp2 (pBabe-Skp2) retrovirus, and used for validation of c-Src as a SCF^Skp2^ substrate. All *in vivo* validation experiments were performed using a technique that preserves the substrate modification and limits co-purification with non-covalently bound modifiers of modified interacting proteins [35]. Briefly, HEK293T cells (ATCC) were lysed under denaturing conditions in 1% SDS (containing 20 mM N-ethyl-maleimide (NEM)) and boiled briefly to disrupt non-covalent interactions, and then the buffer adjusted to 1× RIPA (0.1% SDS, 0.5% NP40, 20 mM NEM, 50 mM Tris (pH 8.5), 150 mM NaCl, 5 mM EDTA, 5 mM NaF, 1 mM sodium orthovanadate, 1 mM PMSF, 2 µg/ml aprotinin, 1 µg/ml pepstatin, and 1 µg/ml leupeptin). The expressed or endogenous putative substrates of ubiquitin, SUMO1, or NEDD8 were then immunoprecipitated from the extracts as indicated. In all cases, immunoprecipitation of extracts with IgG antibodies of the same species served as control.

## Supporting Information

Table S1 Proteins whose ubiquitylation status changed with breast tumor progression. Median values for duplicate proteins spotted on the array were calculated for on-chip ubiquitylation reactions differing only by the addition of low or high grade tumor extract. The proteins are sorted according to a directional measure of fold-change in ubiquitylation status.
(0.98 MB DOC)

Figure S1 Expression level of putative substrates of ubiquitylation that were cloned into Myc- or GST-expression vectors and used in validation experiments. Ten putative substrates of ubiquitylation identified on the protein microarrays but not reported in the literature were selected for validation of the modification in vivo. These ten substrates were cloned into Myc- or GST- expression vectors and were co-expressed with HA-ubiquitin in HEK293T cells. Subsequently, HEK293T cell extracts were prepared using denaturing conditions. Empty vector co-expressed with HA-tagged ubiquitin served as control. Immunoblot, using anti-Myc or anti-GST antibodies, was used to determine the expression level of each substrate which is indicated in each lane as: 1- ADRBK2, 2- ACVR1B, 3- PIM2, 4- PRKCgamma, 5- KIF2C, 6- RPS6KA5, 7- ITK, 8- EPHA1, 9- TRIM52, and 10- EPHA5.
(0.03 MB JPG)
