# Supplementary figures and images for: Development and Validation of a Method for Profiling Post-Translational Modification Activities Using Protein Microarrays
Source: PLoS One. 2010 Jun 28;5(6):e11332. doi: 10.1371/journal.pone.0011332 (PMC2893156; doi:10.1371/journal.pone.0011332)

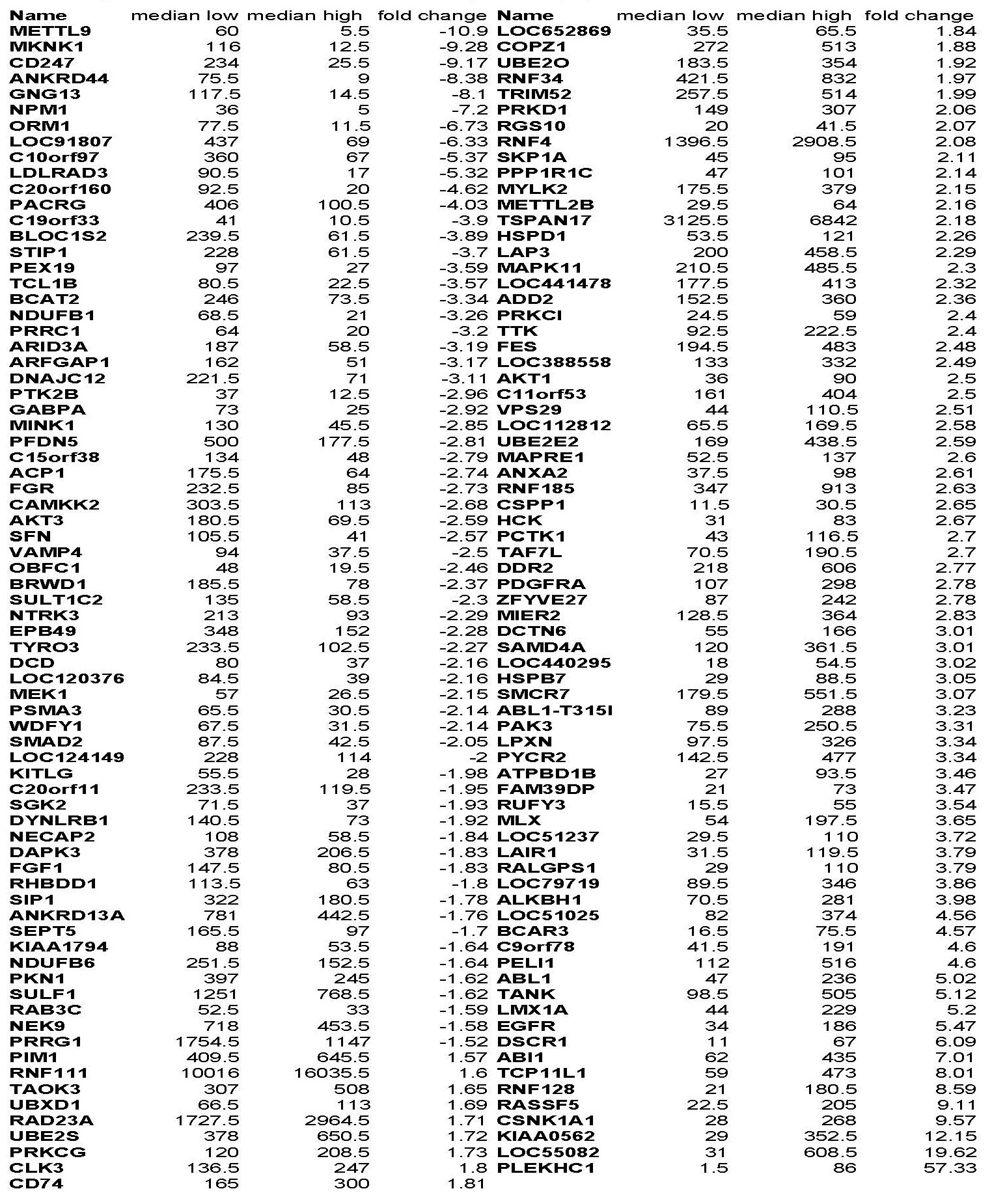

Supplement: Table S1 — Proteins whose ubiquitylation status changed with breast tumor progression. Median values for duplicate proteins spotted on the array were calculated for on-chip ubiquitylation reactions differing only by the addition of low or high grade tumor extract. The proteins are sorted according to a directional measure of fold-change in ubiquitylation status. (0.98 MB DOC) [file pone.0011332.s001.doc]

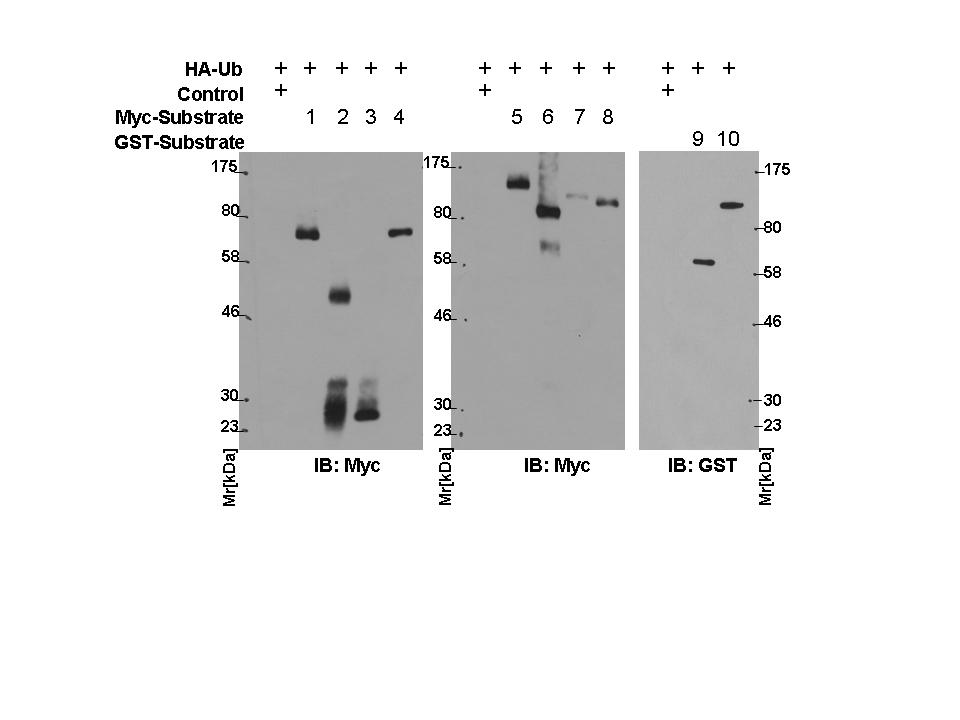

Supplement: Figure S1 — Expression level of putative substrates of ubiquitylation that were cloned into Myc- or GST-expression vectors and used in validation experiments. Ten putative substrates of ubiquitylation identified on the protein microarrays but not reported in the literature were selected for validation of the modification in vivo. These ten substrates were cloned into Myc- or GST- expression vectors and were co-expressed with HA-ubiquitin in HEK293T cells. Subsequently, HEK293T cell extracts were prepared using denaturing conditions. Empty vector co-expressed with HA-tagged ubiquitin served as control. Immunoblot, using anti-Myc or anti-GST antibodies, was used to determine the expression level of each substrate which is indicated in each lane as: 1- ADRBK2, 2- ACVR1B, 3- PIM2, 4- PRKCgamma, 5- KIF2C, 6- RPS6KA5, 7- ITK, 8- EPHA1, 9- TRIM52, and 10- EPHA5. (0.03 MB JPG) [file pone.0011332.s002.jpg]
